# Helminth absence and invasion success of blackchin tilapia (*Sarotherodon melanotheron*) in Thailand

**DOI:** 10.3389/fvets.2025.1529827

**Published:** 2025-02-07

**Authors:** Nannaphat Suwannarat, Alexis Ribas, Jordi Miquel, Srisupaph Poonlaphdecha

**Affiliations:** ^1^Program in Fishery Science and Aquatic Resources, Department of Agricultural Technology, King Mongkut’s Institute of Technology Ladkrabang, Prince of Chumphon Campus, Chumphon, Thailand; ^2^Parasitology Section, Department of Biology, Healthcare and Environment, Faculty of Pharmacy and Food Science, University of Barcelona, Barcelona, Spain; ^3^Institut de Recerca de la Biodiversitat (IRBio), Universitat de Barcelona, Barcelona, Spain

**Keywords:** helminth, invasive fish, enemy release hypothesis, *Sarotherodon melanotheron*, Thailand

## Abstract

**Introduction:**

This study investigates the helminth absence in the invasive blackchin tilapia (*Sarotherodon melanotheron*) in Thailand, testing the Enemy Release Hypothesis (ERH). The ERH suggests that invasive species thrive in new habitats due to the lack of natural parasites that control their populations in native environments. The recent introduction of *S. melanotheron* in Thailand has raised concerns about its ecological and economic impacts.

**Methods:**

We surveyed 164 blackchin tilapia from six different locations in Chumphon Province, Thailand, including the sea, estuary, canal, and shrimp farms, examining them for helminths. Fishermen provided data on the first capture dates in the surveyed areas to determine how long the populations have been established.

**Results:**

No helminths were detected in any of the examined fish. The absence of parasites was consistent across all surveyed environments, suggesting a lack of parasitic burden in the population.

**Discussion:**

The absence of helminths may contribute to the successful expansion of *S. melanotheron* in Thailand, supporting the Enemy Release Hypothesis.

## 1 Introduction

The cichlid fish *Sarotherodon melanotheron* Rüppell 1852 (Cichlidae) is originally distributed in Africa, where it inhabits lagoons and estuaries from Mauritania to the Democratic Republic of the Congo (1). This species has since been introduced to other continents, including America (Suriname and the United States) (1). In Asia, it has been intentionally introduced for aquaculture purposes, particularly in countries such as Cambodia and the Philippines (2–4). In its native Africa, its original distribution range has expanded, demonstrating its capacity to colonize new habitats, such as reservoirs (5). As a euryhaline species, *S. melanotheron* shows high adaptability to various aquatic environments, being found in coastal and lagoon waters as well as freshwater reservoirs in places like Hawaii (6). This includes physiological strategies to protect gills from environmental threats and to maintain protein integrity (7). The reproductive traits of the blackchin tilapia also exhibit significant physiological plasticity, allowing it to adapt to different salinities. This adaptability is crucial for its survival and reproductive success in varying environmental conditions (8).

Furthermore, the introduction of *S. melanotheron* to new regions for aquaculture has had significant economic and ecological impacts. In Asia, for instance, its use in aquaculture has provided a valuable source of protein and income for local communities. However, the introduction of non-native species can also pose risks to local ecosystems, potentially leading to competition with native species and changes in local biodiversity. Overall, the adaptability and resilience of *S. melanotheron* make it a species of significant interest both ecologically and economically. Its ability to thrive in diverse environments and its use in aquaculture underscore its importance, highlighting the need for careful management to mitigate potential ecological impacts.

Tilapias, including the genera *Oreochromis*, *Sarotherodon,* and *Tilapia*, host a rich variety of parasites, many of which have been translocated with their hosts to newly colonized areas (9). The parasites of *Sarotherodon melanotheron* have been well studied in their native distribution areas in Africa, with research conducted in Nigeria (10), Benin (11), and the Ivory Coast (12, 13). These studies have reported a high diversity of parasites found on the skin, gills, lamellae, opercula, and intestines, including several taxonomic groups: Myxosporidia (*Myxobolus*), Monogenea (*Cichlodigyrus*, *Enterogyrus* and *Scutogyrus*), Digenea (*Clinostomum* and *Euclinostomum*), Cestoda (Gryporhynchidae), Acanthocephala (*Acanthogyrus*), Nematoda (*Eustrongylides*), and Copepoda (*Ergasilus* and *Lernaea*) (10–14). Two of the genera reported in Africa, *Clinostomum* and *Eustrongylides*, are considered zoonotic (15, 16). However, data on parasites in introduced populations outside Africa are very limited. For instance, there is no existing data for Thailand, which is our study area. This lack of information poses a challenge for managing the health of introduced populations and impacts of these translocated parasites in new environments. In addition to their ecological significance, wild and farmed tilapias present a potential zoonotic risk, as they can transmit parasitic infections to human populations (17).

In Southeast Asia, *S. melanotheron* was first recorded on scientific literature in open lakes in the Philippines in 2011, and by 2015, it was reported in the coastal waters of Manila Bay (2). The origin of *S. melanotheron*’s introduction in Thailand remains unclear, but it is relatively recent. The first establishment and proliferation were reported in 2002, and by the summer of 2024, the issue was raised in both the Thai parliament and the media due to growing concerns about the negative impacts on aquatic environments and the economy, with estimated losses in the millions of dollars (18). In other regions, such as Hawaii, the introduction of this fish has led to adverse ecological impacts (6). In Thailand, a recent survey in Samut Songkhram Province found that blackchin tilapia was the dominant species at the Don Hoi Lot Ramsar Site (19). In Chumphon Province, our survey area, *S. melanotheron* has spread across all estuaries. Although there is no official report on the introduction of this invasive fish in the area, it appears to be a recent occurrence, as confirmed by the Marine and Coastal Research Center (Central Gulf of Thailand), Mueang Chumphon District, Chumphon Province, through personal communication with one of the authors.

The Enemy Release Hypothesis (ERH) suggests that the abundance or impact of certain non-native species is influenced by the lack of natural enemies in their introduced range compared to their native range, with parasites being one of these natural enemies (20). According to Shinn et al. (9), the translocation of parasites in tilapia species varies based on their life cycles. Trematodes with more complex life cycles, involving multiple hosts, often result in the absence of the same species of first intermediate host (a mollusk), unlike monogeneans, which do not require intermediate hosts. Parasites significantly influence the invasive process of vertebrates by weakening the immune responses of native species, facilitating disease spread, and disrupting ecological balances. This amplifies the impact of invasive vertebrates on local ecosystems (21–24). Understanding the role of parasites in these invasions is crucial for assessing their contribution to invasion success, their potential to introduce novel diseases, and their impacts on native species and ecosystems. This knowledge can inform better management and control strategies. The aim of this study was to investigate the helminth community of this fish species to help explain its high invasive success in Thai aquatic systems. According to the ERH, we expect the blackchin tilapia to exhibit lower helminth diversity in the introduced population studied in Thailand compared to its native distribution areas in Africa.

## 2 Materials and methods

**Figure 1:**
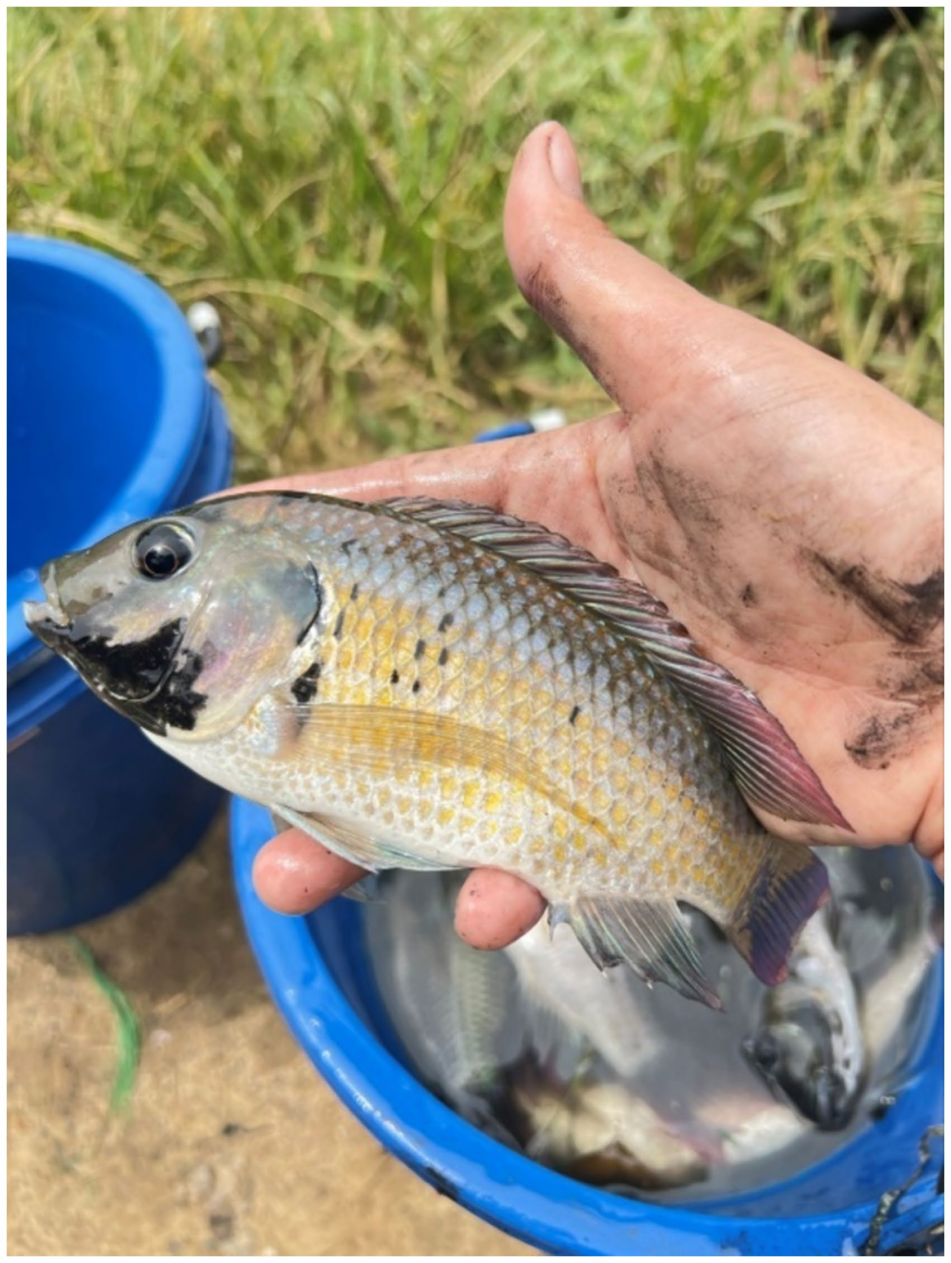
*Sarotherodon melanotheron* from our study area, easily recognized by the distinctive black area on its chin, is readily identified by local fishermen.

**Figure 2:**
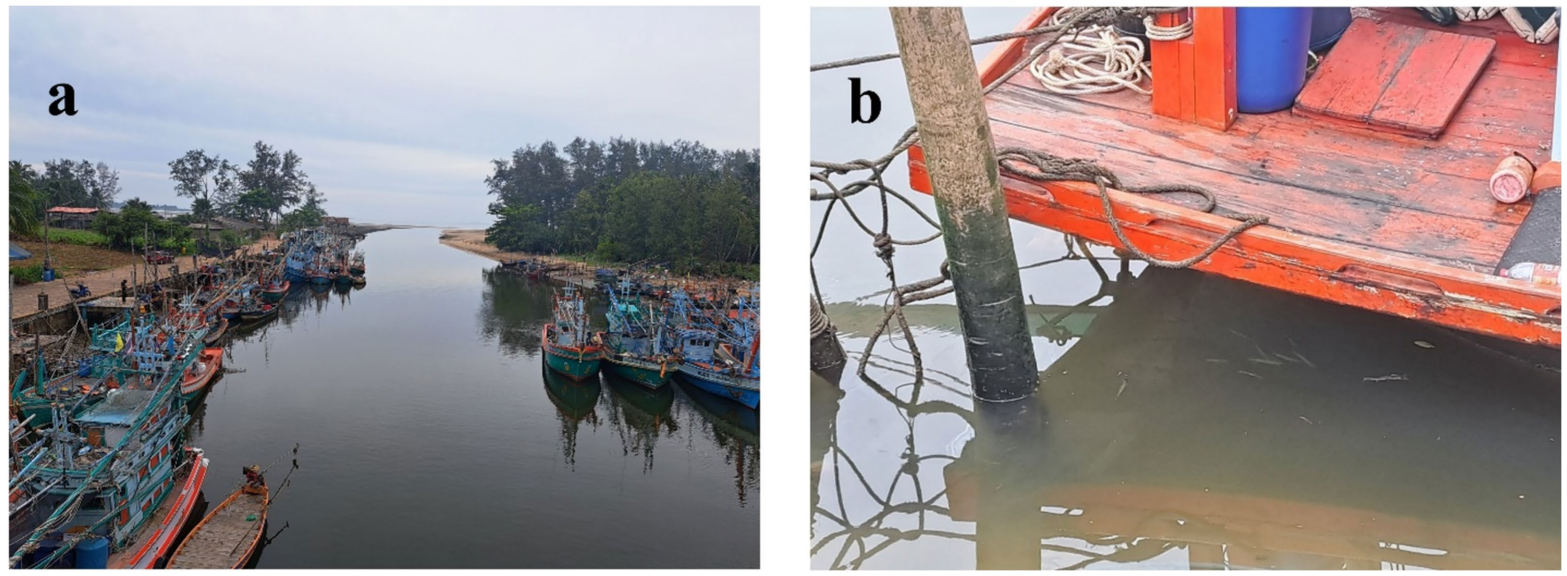
**(A)** One of the representative habitats of *Sarotherodon melanotheron* in the canal, **(B)** blackchin tilapia under a boat in the canal.

**Figure 3:**
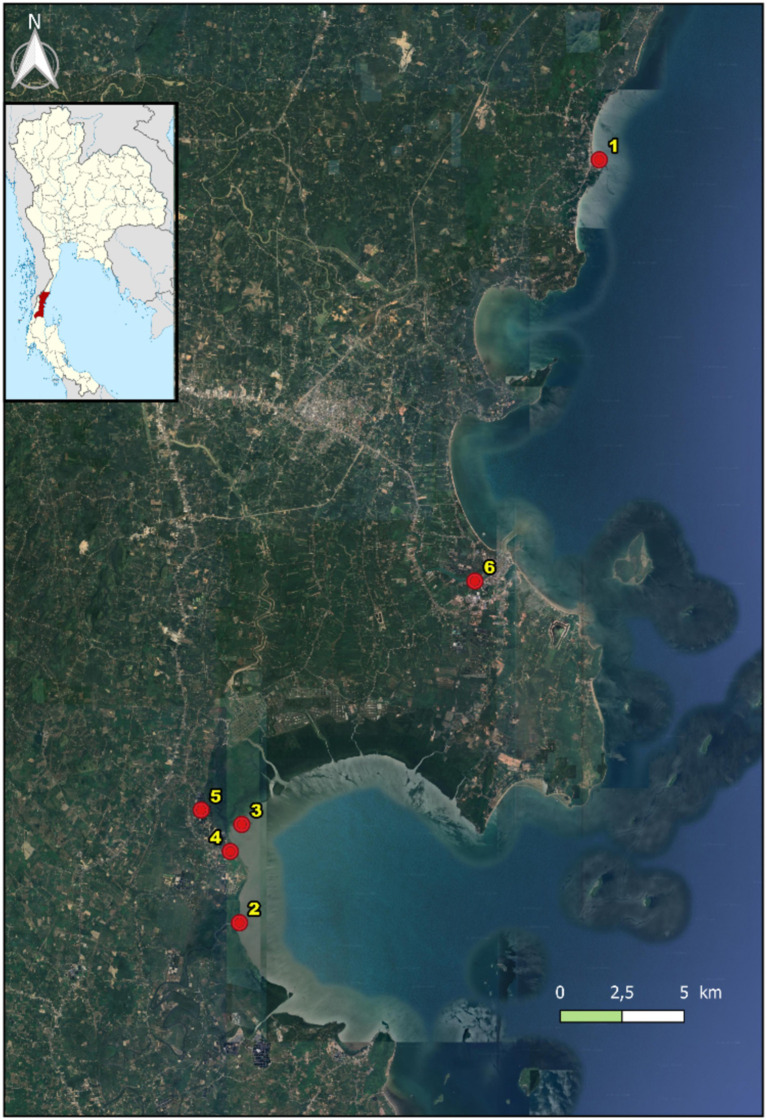
Sampled points of *Sarotherodon melanotheron* in Chumphon Province (province shaded in red on the map of Thailand).

The collecting of blackchin tilapia (Figure 1) was performed from July to August 2024. A total of six points were surveyed in Chumphon Province, covering four different environments: sea, estuary, canal and shrimp farm (Figure 2). The specific locations and details are as follows: (1) Sea (*n* = 30): about 3,000 m from Saphli Pier, Pathio District; coordinates of Saphli Pier: latitude 10.587698, longitude 99.282759. (2) Estuary of canal (*n* = 23): Pak Phraek, Sawi District; coordinates: latitude 10.311691, longitude 99.150417. (3) Sea in front of estuary of canal (*n* = 20): Thung Kha, Mueang Chumphon District; coordinates: latitude 10.347286, longitude 99.151261. (4) Shrimp farm 1 (*n* = 25): Wisai Nuea, Mueang Chumphon District; coordinates: latitude 10.337490, longitude 99.147035. (5) Shrimp farm 2 (*n* = 30): Wisai Nuea, Mueang Chumphon District; coordinates: latitude 10.352470, longitude 99.136356. (6) Canal (approximately 3,000 m from the mouth to the sea) (*n* = 36): Pak Nam, Mueang Chumphon District; coordinates: latitude 10.435289, longitude 99.23701 (Figure 3). Local fishermen were advised to contact to the University when blackchin tilapia were caught during daily fishing activities. Upon notification, the fish were collected and transported to the laboratory for immediate dissection. The survey for helminths was conducted on the skin surface, fins, mouth, liver, body cavities and gills using a stereomicroscope. The gastrointestinal tract was examined following the method described by Justine et al. (25). All individuals were photographed, weighed, and measured from head to tail fork. To assess the arrival data of the blackchin tilapia, 10 fishermen at each locality were asked, “In which year did you first catch blackchin tilapia in this area?” If there was no consensus among them, the oldest reported date was considered valid. Salinity was determined using a Master Refractometer (Atago, Japan) in October 2024. This project was reviewed and approved by the Animal Care and Use Committee of King Mongkut’s Institute of Technology Ladkrabang, Thailand, under approval number ACUC–KMITL-RES/2024/012.

## 3 Results

A total of 164 blackchin tilapia were examined, ranging in size from 65 to 250 mm, with an average length of 158.61 mm. The fish weighed between 4.9 and 239.4 g, with an average weight of 92.76 g. Of these, 80 were female, 80 were male, and 4 were undetermined sex. No helmints were detected in any of the studied organs. The arrival of the blackchin tilapia was estimated as follows: (1) Sea: about 3,000 m from Saphli Pier, Pathio District: 2019; (2) Estuary of canal: Pak Phraek, Sawi District: 2018; (3) Sea in front of estuary of canal: Thung Kha, Mueang Chumphon District: 2018; (4) Shrimp farm 1: Wisai Nuea, Mueang Chumphon District: 2019; (5) Shrimp farm 2: Wisai Nuea, Mueang Chumphon District: 2019; (6) Canal (approximately 3,000 m from the mouth to the sea): Pak Nam, Mueang Chumphon District: 2016. Salinity varied across the different surveyed habitats as follows: (1) 32–33 ppt; (2) 32–33 ppt; (3) 32–33 ppt; (4) 15–20 ppt; (5) 15–20 ppt; and (6) 15–20 ppt.

## 4 Discussion

To our knowledge, this is the first survey of helminths in an introduced population of *Sarotherodon melanotheron* in Thailand, and it may be extended to Southeast Asia [as reviewed by Acosta-Pérez et al. (17)]. Our research tested the Enemy Release Hypothesis (ERH), which suggests that alien species experience greater invasion success in new habitats because they are free from the natural enemies, such as parasites, that regulate their populations in their native environments. This absence of parasites allows invasive species to outcompete native species. Introduced species often originate from relatively small subsets of their native populations, which reduces the likelihood of introducing parasites. Additionally, if the newly colonized area lacks the necessary intermediate hosts, parasites cannot become established (20, 26). In the present study, the absence of parasites is notable, including monogeneans of African origin that have spread to new geographical areas, such as Mexico in *Oreochromis niloticus* (27). In China (28) also reported nine monogenean species infecting cultured tilapia. Similarly, in Madagascar, a subset of dactylogyridean monogeneans arrived from continental Africa with invasive fish (29). In Panama’s Canal watershed, *O. niloticus* was infected by a single parasite (monogenean) species from its native range (30). Our findings align with Firmat et al. (31), who documented the complete loss of gill parasites in the invasive cichlid *O. mossambicus* in New Caledonia. A key factor to consider is the time since these populations were established. In the study by Jiménez-Sánchez et al. (32), the populations had been established for approximately 30 years, whereas in our study, the introduction is much more recent, between four and 8 years depending on the locality. The importance of colonization time has been demonstrated in other cases as well. For example, the dataset of 32 British freshwater fish species and their helminth parasites was analyzed, including seven introduced host species. The analysis concluded that helminth species richness is correlated with the time since the fish hosts were introduced to Britain (33). Also, it has been shown that the helminth parasite diversity of the Eurasian round goby (*Neogobius melanostomus*) introduced in Canada doubled after 15 years. The authors reached this conclusion by comparing the original study, conducted soon after the establishment of this invasive fish, with a resampling conducted 15 years later using the same sampling design (34, 35, 36). These findings suggest that the recent introduction of *S. melanotheron* in Thailand may explain the absence of helminths in our survey conducted at the invasion front. The lack of helminths could be attributed to the short time since introduction, which may not have allowed sufficient time for the establishment and spread of parasites. Continued monitoring and further studies are necessary to understand the long-term dynamics of parasite–host interactions in these newly introduced populations.

Further studies are necessary to evaluate the potential advantages that cichlids might gain in the invasive areas when infected with a low richness and diversity of helminths (32). The absence of parasites in this population could be significant, and examining sympatric fish species will be relevant for further understanding. A limitation of our study is the lack of a survey for metacercariae in muscle tissue using a digestion protocol due to logistical constraints, a gap that future research should address. In addition to the ecological negative effect, fish-borne zoonotic trematodes, including heterophyids and opisthorchiids, are widespread in native and farmed fish in Southeast Asia (35). Data on the susceptibility of *S. melanotheron* to these zoonotic trematodes in natural populations would be valuable. An experimental study by Kopolrat et al. (35) under controlled conditions examined the susceptibility to *Haplorchis taichui* in five aquaculture fish species from Thailand, showing susceptibility variations in the success of parasitization. Highly susceptible fish species included *Barbonymus gonionotus, Cyprinus carpio*, and *Cirrhinus mrigala*, with values of 93.33 and 100% after exposure to cercariae. In contrast, cichlids such as Nile tilapia (*O. niloticus*) and red tilapia (*Oreochromis aureus* x *Oreochromis mossambicus*) showed no susceptibility. A study in Thailand on translocated Nile tilapia found three zoonotic species: *Stellantchasmus falcatus*, *Haplorchis pumilio*, and *Procerovum* var*ium* (36). It should be noted that this fish has been present in Thailand for decades, making it an incomparable model to newly introduced species. Additionally, blackchin tilapia is not commonly valued as a food resource, so the potential transmission to these trematodes to humans should not be a major concern. However, the normalization of this fish in Thai ecosystems could eventually lead to its introduction as a food resource, potentially raising food safety issues.

In conclusion, the absence of helminths at the invasion front of blackchin tilapia may partly explain the successful expansion of this invasive species. This lack of parasitic burden could provide a competitive advantage, facilitating its spread and establishment in new environments.

## Data Availability

The raw data supporting the conclusions of this article will be made available by the authors, without undue reservation.
